# Challenges in early diagnosis of cancer: the fast track

**DOI:** 10.1080/02813432.2020.1794415

**Published:** 2020-08-14

**Authors:** Knut Holtedahl

**Affiliations:** Department of Community Medicine, UiT The Arctic University of Norway, Breivika, Norway

In recent years, many countries have introduced a cancer patient pathway (CPP), often called a ‘fast track’. It is intended to shorten the time interval between consultation and treatment in cases of suspected cancer [1]. In several of the countries, this goal has been achieved for referred patients, which is in itself positive. A higher survival rate seems to be within reach. For five common cancers, a shortening of the longest diagnostic intervals may be linked to a higher five-year survival rate [2].

GPs interact with patients and interpret the presented symptoms, but linking interpreted symptoms to CPPs is complex [3]. The probability of fast-tracking is higher for patients manifesting alarm symptoms; however, fewer than one-half of undiagnosed cancer patients present an alarming symptom in consultations [4]. Fast-track referrals are less likely when symptoms are non-specific and in patients belonging to low incidence demographics [5]. GPs encounter many cases where patients have vague symptoms and where further examination fails to rule out the possibility of cancer. One study found that 7% of cancer patients displayed only ‘low-risk-but not-no-risk’ symptoms [6]. Some potential cancer symptoms are very prevalent in the general population [7]. Achieving a balance between doing the necessary and avoiding the unnecessary poses dilemmas for GPs.

There is extensive primary care evidence of symptoms with a greater incidence in undiagnosed cancer patients than in patients in general [8]. During the consultation, it can be favorable for a GP to think about and attempt to quantify the most central *parameters* suggesting the *associations* between symptom and cancer: Sensitivity, that is, the proportion of undiagnosed cancer patients who presents the symptom in the consultation, and Specificity, that is, the proportion of consulting non-cancer patients who do not present the symptom in question.

Cancer patients experience symptoms sooner or later. However, at the initial consultation, *sensitivity* for a single symptom in relation to cancer is low, more often below 10% than above it [4]. In other words, cancer cannot be ruled out even if alarm symptoms are absent. Also, the common absence of an alarming symptom in early cancer complicates referrals through fast track. *Specificity* is often high in secondary care, where endoscopies and imaging facilitate final diagnostic decisions, or at least admission onto the fast track. In general practice, symptoms that are rare in non-cancer patients should always be investigated until they can be explained. One example is abnormal bleeding from body orifices, with a specificity of 99% to cancer [4].

It is not infrequent for GPs to have to consider the possibility of cancer at the end of a consultation. Alas, some symptoms are common in both benign and malignant diseases. Therefore, single symptoms can have low *positive predictive values* (PPV). Most probabilities are below 2–3% for cancer, and rarely above 5% [9]. However, guidelines from the UK’s National Institute for Health and Care Excellence (NICE) recommend that GPs initiate rapid follow-up of suspected cancer from a probability as low as 3% [10]. Combining a symptom with positive test results or relevant clinical findings tends to increase the PPV [8]. For a GP, this makes it possible to revise the PPV based on the information available for individual patients, while still complying with the NICE threshold [11].

Clinical competence means the ability to professionally interpret symptom presentations. While some phenomena may be too subtle to quantify, they may still influence rational decisions. Norwegian GPs were interviewed on how the idea of cancer might arise in a general practice consultation [12]. The GPs’ experiences included the application of basic knowledge, interpersonal awareness, fear of cancer and intuition. Intuition was described as a tacit feeling of alarm that could be difficult to verbalize but was nevertheless helpful [12]. In another study, intuition-based cancer suspicions could be associated with subsequent cancer diagnoses [13]. Such observations, both qualitative and quantitative, may contribute to demystifying intuition and giving it a natural place in the diagnostic reasoning of GPs.

An early cancer diagnosis is one of the emotionally and intellectually engaging challenges of general practice. Fast-track referral should be the choice if the GP’s cancer suspicion – whether due to findings and/or intuition – is strong or persistent; sometimes regardless of the presented symptoms. Primary care remains an important area for research.
